# Conditions associated with worse acceptance of a simplified accelerated recovery after surgery protocol in laparoscopic colorectal surgery

**DOI:** 10.1186/s12893-021-01206-2

**Published:** 2021-05-03

**Authors:** Fábio Lopes de Queiroz, Antonio Lacerda-Filho, Adriana Cherem Alves, Fábio Henrique de Oliveira, Paulo Rocha França Neto, Rodrigo de Almeida Paiva

**Affiliations:** 1 Colorectal Surgery Department, Hospital Felicio Rocho, Rua Felipe Dos Santos, 760, 501-3, Belo Horizonte, Minas Gerais CEP 30180160 Brazil; 2 Department of Surgery at the School of Medicine, Federal University of Minas Gerais, Belo Horizonte, Brazil; 3 School of Medicine, Federal University of Minas Gerais, Belo Horizonte, Brazil; 4 Semper Hospital- Belo Horizonte, Belo Horizonte, Brazil; 5 Department of Surgery, Federal University of São João del Rey, Divinópolis, Brazil; 6 Hospital FelicioRocho, Belo Horizonte, Brazil

**Keywords:** Colectomy, Accelerated postoperative recovery, Perioperative care, Nutrition

## Abstract

**Background:**

Enhanced Recovery Surgical Programs were initially applied to colorectal procedures and used as multimodal approach to relieve the response to surgical stress. An important factor that negatively impacts the success of these programs is the poor tolerance of these patients to certain items in the adopted protocol, especially with regard to post-operative measures. The identification of these factors may help to increase the success rate of such programs, ensuring that benefits reach a greater number of patients and that resources are better allocated. Thus, the aims of this study were to assess the results of the implementation of a Simplified Accelerated Recovery Protocol (SARP) and to identify possible factors associated with failure to implement postoperative protocol measures in patients submitted to laparoscopic colorectal surgery.

**Methods:**

161 patients were randomly divided into two groups. The SARP group (n = 84) was submitted to the accelerated recovery program and the CC group (n = 77), to conventional postoperative care. The SARP group was further divided into two subgroups: patients who tolerated the protocol (n = 51) and those who did not (n = 33), in order to analyze factors contributing to protocol nontolerance.

**Results:**

The groups had similar sociodemographic and clinical characteristics. The SARP group had a shorter hospital stay, better elimination of flatus, was able to walk and to tolerate a diet sooner (*p* < 0.0001). Complications rates and readmissions to emergency room were similar between groups. Multivariate analysis revealed that prolonged operating time, stoma creation and rates of surgical complications were independently associated with poor adherence to SARP (p < 0.0001).

**Conclusions:**

The use of our SARP resulted in improved recovery from laparoscopic colorectal surgery and proved to be safe for patients. Extensive surgeries, occurrence of complications, and the need for ostomy were variables associated with poor program adhesion.

*Trial registration* Trial Registry: RBR2b4fyr—Date of registration: 03 October 2017.

## Background

Enhanced Recovery After Surgery™ (ERAS) programs aim to replace traditional perioperative practices, which have long been adopted and used, with interventions that have proven to be beneficial, based on solid scientific evidence. ERAS programs were initially applied to colorectal surgery and used a multimodal care approach to relieve the response to surgical stress, accelerating postoperative recovery and reducing costs for both public and private health systems [1, 2]. However, in spite of their scientifically-proven advantages and of the widespread adoption of minimally-invasive techniques, one their main features, ERAS™ protocols are used in less than one-third of surgical procedures in the United States and the United Kingdom [2–5]. This is mainly due to operational difficulties, initial costs for implementation and also due to the complexity and a number of interventions advocated by most of the described protocols, which hampers patients acceptance making it difficult to be adopted by many institutions [6, 7].

The adoption of simplified protocols, with a smaller number of interventions, could favor the acceptance of such measures not only by patients, but also by a greater number of institutions, mainly in developing countries, where resources are scarce. However, even with the use of less complex protocols a significant percentage of patients did not tolerate the implemented measures. This is true, especially regarding to post-operative measures, which have a non-acceptance rate of up to 40% [5–8], reducing substantially the expected benefits of adopting the same. The identification of factors related to non-tolerance may help to increase the success rate of such programs, ensuring that benefits reach a greater number of patients and that resources are better allocated. Thus, the aims of this study were to assess the results of the implementation of a Simplified Accelerated Recovery Protocol (SARP) and to identify possible factors associated with failure to implement postoperative protocol measures in patients submitted to laparoscopic colorectal surgery.

## Methods

This was a prospective randomized study comparing two groups of patients. One was submitted to simplified accelerated recovery protocol (SARP), focusing on postoperative measures (Table 1), while the other group underwent conventional postoperative care (CC) (Table 2). Patients over 18 years of age were included, as per classifications I and II of the American Society of Anesthesiologists (ASA). These patients underwent elective laparoscopic colorectal surgery with anastomosis, with or without protective ostomy. The study excluded patients with end-stage colorectal cancer, mental or psychiatric disorders, and those with contraindications to laparoscopic surgery. Also excluded were pregnant patients and those who had previously undergone esophageal, gastroduodenal, or pancreatic surgery, as well as those who refused to participate in the study. All participants signed a statement of Informed Consent.

**Table 1:** Simplified accelerated postoperative recovery program (SARP)

1. Removal of the nasogastric catheter prior to extubation
2. Restricted liquid diet approximately 8 h postoperatively, soft diet on the 1st POD, and free diet on the 2nd POD
3. Suspension of serum therapy on the 1st POD, with maintenance of the salinized venous catheter
4. Early mobilization, starting 6–8 h postoperatively, with assistance of the physiotherapy team
5. Food quantification performed by the nutrition team
6. Removal of the urinary catheter within 24 h postoperatively
7. Removal of abdominal drains within 48 h postoperatively

**Table 2:** Conventional postoperative care (CC)

1. Maintenance of the nasogastric catheter until effective peristalsis or reduction of flow
2. Restricted liquid diet initiated only after elimination of gas and/or presence of bruit
3. Daily gradual progression of oral diet
4. Removal of drains after 4 days, if there were no signs of complications
5. Removal of the urinary catheter within 48 h postoperatively
6. Maintenance of serum therapy until adequate acceptance of a soft diet
7. Mobilization, according to the patient’s request

**Fig. 1:**
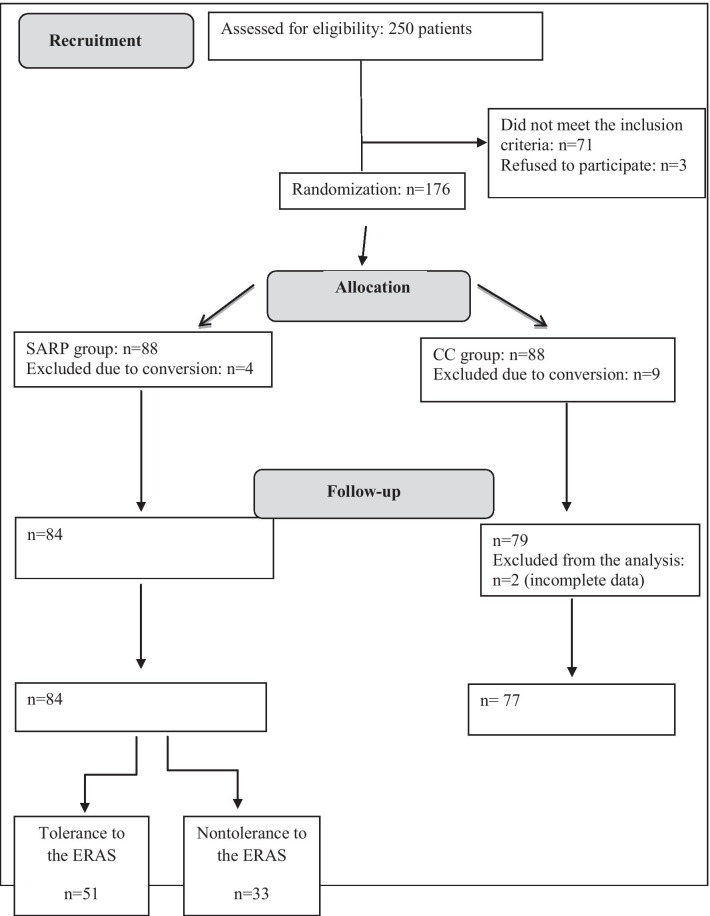
CONSORT diagram of patient allocation

The patients underwent surgery at the Colorectal Surgery Department of Felício Rocho Hospital, a tertiary referral hospital in Belo Horizonte, Brazil, over a two-year period. Initially, 250 patients were considered eligible; 71 patients were excluded for not meeting the inclusion criteria, while three were excluded for refusing to participate in the study. Of the 176 participating patients who underwent colorectal resections by video-assisted laparoscopy, 13 (7.4%) and 2 (1.1%) patients were excluded due to conversion and data collection failures, respectively. The remaining 161 patients were included in the data analysis. The distribution of patients and of analyzed groups is presented in the CONSORT diagram [9] (Fig. 1).

Colon preparation was performed with oral sodium phosphate. A regular diet was maintained until eight hours before surgery and patients were encouraged to ingest clear liquids up to three hours before surgery. All patients received standardized anesthetic care and antimicrobial prophylaxis was administered in a single dose of ceftriaxone 2 g and metronidazole 1.5 g, thirty minutes before skin incision. Urinary and nasogastric catheters were placed at the start of surgery to monitor urine output and to achieve gastric decompression, respectively.

All procedures were started using video-assisted laparoscopy, with two 10 mm portals and three 5 mm portals. Creation of larger than necessary longitudinal or transverse incisions, to allow for the removal of surgical specimens (> 8 cm) was considered to be a conversion. A diverting loop ileostomy was created for all patients undergoing low anterior resection.

At the end of the surgery, the patients underwent simple randomization by drawing numbers 1 (SARP) or 2 (CC) marked on cards in a closed box. Groups were compared to assess their similarities and then they were compared with each other to prove the benefits of the simplified protocol of accelerated recovery. In this case, hospital discharge until the third postoperative day (POD) was considered as a criterion for tolerance to SARP [10–12]. Patients who were not discharged by the third POD, according to these criteria, were considered intolerant to the program. The established criteria for discharge were stable vital signs, an alert and oriented mental status, absence of symptoms and of suspected complications, unassisted walking, tolerance to a soft or solid diet, elimination of flatus, spontaneous diuresis, good pain control with oral analgesics, self-sufficiency in basic daily activities, and the patient’s expressed desire to go home.

In order to assess possible factors working against the adoption of the protocol in the SARP group, the “tolerant” subgroup (patients discharged by the third POD) and the “non-tolerant” subgroup (those discharged after the third POD) were compared to each other. The variables potentially involved in tolerance or non-tolerance to program items were presence of complications, need for ostomy, operative time, body mass index (BMI), age, sex, ASA classification 1 or 2, presence of comorbidities, malignant or benign disease, and type of surgery. Regarding the type of surgery, two groups were formed: patients who underwent surgery involving only the colon (colectomies) and those who underwent surgeries that involved rectal resection that is total or partial mesorectal excison.

Upon discharge, oral and antispasmodic analgesics (hyoscine and dipyrone or paracetamol) were prescribed to the patients, and the use of enoxaparin at 40 mg/day for four weeks was recommended. Patients were guided on how to contact the team in case of intercurrent events, such as abdominal pain or distension, fever, an interruption in the elimination of flatus and feces, among others. All patients were instructed to return for postoperative consultation seven to ten days after discharge, or as needed.

Postoperative complications were considered to be those that developed by the thirtieth POD, categorized according to the Dindo–Clavien classification [13].

This study was approved by the Research Ethics Committee of our institution and by the Research Ethics Committee of the Federal University of Minas Gerais. It was registered on the Brazilian Platform of Research under CAAE number 43719015.4.0000.5149. It is enrolled in the Brazilian Clinical Trials Registry, linked to the International Clinical Trials Registry of the World Health Organization under number RBR-2b4fyr.

### Statistical analysis

The sample size was 161 determined using Epi Info software version 7.2, with power equal to 0.80, a margin of error of 5,02%, an expected tolerance percentage of 0.12, and a confidence level of 95%. The statistical analysis of the variables was performed using the SPSS program (IBM—version 20.0, 2011). To compare the quantitative measures, the Mann–Whitney U test was used for non-normal distributions, and Student’s t-test for normal distributions, with verification by the Shapiro–Wilk test. Pearson’s asymptotic and exact chi-square tests were used to compare category variables. Multivariate logistic regression was used to analyze association. Variables with an association at the 0.20 level were considered candidates for the multivariate model. They were jointly analyzed until only those with significance at the 0.05 level remained. The quality of the adjustment was analyzed using the Hosmer–Lemeshow test. A *p*-value < 0.05 was considered statistically significant.

## Results

Of the 161 patients included in the final analysis, 84 (52.2%) were allocated to the SARP group and 77 (47.8%), to the CC group. Ninety-six patients were female (59.6%). Age ranged from 25 to 95 years, with an average of 57.4 ± 12.6 years. BMI ranged from 18.0 to 51.0 kg/m^2^, with a median of 25.5 kg/m^2^.

The two groups were similar with respect to demographic characteristics, as well as to the type and to the duration of the surgery performed, as shown in Table 3.

**Table 3:** Demographic, clinical, and surgical characteristics of patients in the SARP and CC groups (n = 161)

Variables	SARP (n = 84)	CC (n = 77)	*p*-value
*Age*			
Mean ± SD	55.01 ± 12.60	58.39 ± 13.86	0.074^a^
Median (Q1; Q3)	55.5 (48.0; 61.8)	58.0 (51.0; 70.0)	
*Sex, n (%)*			
Female	51 (53.1)	45 (46.9)	0.769^b^
Male	33 (50.8)	32 (49.2)	
*BMI*			
Mean ± SD	25.41 ± 5.14	25.89 ± 4.91	0.890^a^
Median (Q1; Q3)	25.47 (22.54; 28.41)	25.24 (22.60; 27.97)	
*ASA, n (%)*			
1	41 (51.9)	38 (48.1)	0.945^b^
2	43 (52.4)	39 (47.6)	
*Disease, n (%)*			
Malignant	57 (49.6)	58 (50.4)	0.295^b^
Benign	27 (58.7)	19 (41.3)	
*Type of surgery, n (%)*			
Colectomy	70 (83.33)	59 (76.62)	0.084^b^
Proctectomy	14 (16.67)	18 (23.28)	
Duration of the surgery, min	234.25 ± 67.11	249.81 ± 77.34	
Mean ± SD	240.00 (180.00; 270.00)	240.00 (195.00; 300.00)	
Median (Q1; Q3)			0.274^a^
*Concomitant resection of other organs, n (%)*			
Yes	25 (50.0)	25 (50.0)	0.711^b^
No	59 (53.2)	52 (46.8)	
*Need for ostomy, n (%)*			
Ileostomy	14 (37.8)	23 (62.2)	0.061^c^
No	69 (56.1)	54 (43.9)	

*SARP* simplified accelerated postoperative recovery program, *CC* conventional postoperative care, *BMI* body mass index, *SD* standard deviation, *Q1; Q3* interquartile range

^a^Mann Whitney U test; ^b^asymptotic chi-square test; ^c^exact chi-square test

The median time of surgical procedures was 240.0 min and did not differ between groups (*p* = 0.274). The most common surgical indication was colorectal cancer, with 115 cases (71.4%). Thirty-seven patients (23%) required ostomy.

A total of 39 patients developed complications, with an overall rate of 24.2%. The SARP group presented a rate of 25% (21/84), while the CC group presented a rate of 23.4% (18/77) (*p* = 0.810). One patient in the SARP group died on the sixth POD due to an anastomotic fistula, resulting in an overall mortality rate of 0.62%. Four patients, with three belonging to the CC group, returned to the emergency room after discharge and were readmitted to the hospital. One patient presented with an anastomotic fistula, one with dehydration, and two with nausea and vomiting caused by an adynamic ileus. The median length of hospital stay was 3.0 days in the SARP group and 5.0 days in the CC group (*p* < 0.0001). In addition to a shorter hospitalization period, patients in the SARP group started walking, expelling gas, and tolerating a diet sooner. On the other hand, patients in this group relied more heavily on analgesics (Table 4).

**Table 4:** Comparative analysis of postoperative results among patients in the SARP and CC groups (n = 161)

Variables	SARP (n = 84)	CC (n = 77)	*p*-value
	n %	n %	
*Hospitalization time*			
Median (Q1; Q3)	3.00 (2.0; 4.00)	5.00 (5.0; 7.00)	** < 0**.**0001**^**a**^
*Hospital discharge*			
≤ 3 days	51 (60%)	0 (0.0)	** < 0**.**0001**^**b**^
> 3 days	33 (40%)	77 (100%)	
*Nausea*			
Yes	30 (44.8)	37 (55.2)	0.113^b^
No	54 (57.4)	40 (42.6)	
*Vomiting*			
Yes	19 (46.3)	22 (53.7)	0.387^b^
No	65 (54.2)	55 (45.8)	
*Walking (days)*			
1st POD	64 (65.3)	34 (34.7)	** < 0**.**0001**^**b**^
After 1st POD	20 (31.7)	43 (68.3)	
*Elimination of gas (days)*			
1st POD	49 (65.3)	26 (34.7)	**0.002**^**b**^
After 1st POD	35 (40.7)	51 (59.3)	
*Bowel movements*			
Up to 3rd POD	56 (52.8)	50 (47.2)	0.817^b^
After 3rd POD	28 (50.9)	27 (49.1)	
*Tolerance to free diet (days)*			
Up to 3rd POD	72 (97.3)	2 (2.7)	** < 0**.**0001**^b^
After 3rd POD	12 (13.8)	75 (86.2)	
*Use of analgesics, anti-inflammatory drugs, and opioids*			
Yes	82 (54.7)	68 (45.3)	**0.019**^**b**^
No	2 (18.2)	9 (81.8)	
*Return to the hospital and rehospitalization after discharge*			
Yes	1 (25.0)	3 (75.0)	0.350^c^
No	83 (52.9)	74 (47.1)	
*Postoperative complications (Clavien–Dindo)*			
No complications	63 (51.6)	59 (48.37)	0.810
Complication, CD ≥ 1	21 (53.8)	18 (46.2)	

Bold values indicate better results in patients submitted to the SARP protocol

*SARP* simplified accelerated recovery program, *C* conventional postoperative care, *CD* Clavien–Dindo, *POD* postoperative day

^a^Mann–Whitney U test; ^b^asymptotic chi-square test; ^c^exact chi-square test

In the SARP group, 51 of 84 patients were discharged by the third POD, resulting in a success rate of 60% for the implementation of SARP, according to the criteria adopted in the present study (Table 4).

When comparing the two subgroups in the SARP group, (i.e., patients who tolerated and those who did not tolerate the protocol), univariate analysis demonstrated that the development of complications, the need for ostomy, a longer operative time, and the performance of rectal resection were all factors associated with nontolerance to SARP (Table 5).

**Table 5:** Results of the univariate analysis of the possible variables that influenced the tolerance or nontolerance to the accelerated recovery program in the subgroups of the SARP group (n = 84)

Variables	Tolerance to “SARP”	Nontolerance to “SARP”	*p*-value
	(n = 51)	(n = 33)	
*Postoperative complications (Clavien–Dindo), n (%)*			
Yes, CD ≥ 1	4 (19.0)	17 (81)	** < 0**.**0001**^**b**^
No	47 (74.6)	16 (25.4)	
*Need to perform ostomy, n (%)*			
Yes	2 (14.3)	12 (85.7)	** < 0**.**0001**^**b**^
No	49 (70)	21 (30.0)	
*Duration of surgery, min*			**0.004**^**d**^
Mean ± SD	217.87 ± 56.86	262.00 ± 75.89	
Median (Q1; Q3)	252.50 (210.00; 305.00)	210.00 (180.00; 240.00)	
*Type of surgery, n (%)*			
Colectomy	49 (70.0)	21 (30.0)	
Proctectomy	2 (14.3)	12 (85.7)	** < 0**.**0001**^**b**^
*BMI, n (%)*			
< 25	14 (50)	14 (50)	0.069^b^
≥ 25	30 (71.4)	12 (28.6)	
*BMI, n (%)*			
Eutrophic	13 (46.4)	14 (53.6)	0.064^b^
Overweight	20 (70.7)	10 (29.3)	
Obese	9 (90)	1 (10)	
*Age, n (%)*			
< 65 years	45 (65.2)	24 (34.8)	0.566^b^
≥ 65 years	8 (57.1)	6 (42.9)	
*Age range, n (%)*			
< 40 years	6 (66.7)	3 (33.3)	0.406^c^
40 to 60 years	32 (62.7)	19 (37.3)	
60 to 70 years	10 (66.7)	5 (33.3)	
> 70 years	3 (33.3)	6 (66.7)	
*Sex, n (%)*			
Female	32 (62.7)	19 (37.3)	0.790^a^
Male	21 (65.6)	11 (34.4)	
*ASA classification, n (%)*			
1	24 (60.0)	16 (40.0)	0.481^a^
2	29 (67.4)	14 (32.6)	
*Presence of comorbidity, n (%)*			
Yes	33 (63.5)	19 (36.5)	0.923^b^
No	20 (64.5)	11 (35.5)	
*Disease, n (%)*			
Malignant	31 (54.4)	26 (45.6)	0.084^b^
Benign	20 (74.1)	7 (25.9)	
*Surgical time, n (%)*			
≤ 3	0 (0.0)	0 (0.0)	
> 3	51 (60.7)	33 (39.3)	–
*ICU time, n (%)*			
Up to 1 day	46 (62.2)	28 (37.8)	0.477^c^
More than 1 day	7 (77.8)	2 (22.2)	
*Use of opioids, n (%)*			
Yes	41 (62.1)	25 (37.9)	0.517^b^
No	12 (70.6)	5 (29.4)	

Bold values indicate factors that negatively influenced the acceptance of SARP, in the univariate analysis

*SARP* simplified accelerated postoperative recovery program, *RC* right colectomy, *TME* total mesorectal excision, *CD* Clavien–Dindo, *SD* standard deviation, *Q1; Q3* interquartile interval, *ICU* intensive care unit, *ASA* American Society of Anesthesiology

*Pearson’s chi-square test

^a^Mann-Whitney U test; ^b^asymptotic chi-square test; ^c^exact chi-square test; ^d^Student’s t-test

In multivariate analysis, the development of complications, a longer operative time, and the need for ostomy were identified as predictors of non-tolerance to SARP items (Table 6).

**Table 6:** Results of the multivariate analysis of the variables that influenced the tolerance or nontolerance of the SARP items (n = 84)

Variables	OR (95% CI OR)	P-value
Type of surgery	2.74 (0.06; 121.57)	0.602
Complications (Clavien–Dindo)	14.78 (3.42; 63.93)	** < 0**.**0001**
Surgical time	1.01 (1.002; 1.021)	**0**.**015**
Need for ostomy	11.20 (1.65; 75.89)	**0**.**0013**
Age	1.04 (0.98; 1.09)	0.181
Sex	1.84 (0.51; 6.68)	0.356
Neoplasm	1.25 (0.31; 4.99)	0.757

Bold values indicate factors that negatively influenced the acceptance of SARP, in the multivariate analysis

Hosmer–Lemeshow test (p = 0.930). Adjustment quality test of the logistic regression model

*SARP* simplified accelerated postoperative recovery program

## Discussion

The simplified accelerated recovery protocol used in this study showed benefits compared to a conventional care protocol, with a median length of hospital stay of three days in the SARP group, significantly lower, than in the conventional group. Furthermore, patients passed gas and tolerated the prescribed diet sooner.

The results obtained with regard to the conversion rate, morbidity, and mortality are all comparable to those of other studies [14, 15].Complication and readmission rates, as well as returns to the emergency room, were not different between groups. Therefore, we conclude that the implementation of an accelerated recovery protocol, even if simplified (SARP), was beneficial and safe for patients, as it shortened hospital stays without increasing the risk of complications or rehospitalizations [10, 16].

In addition, the present study was able to demonstrate a 60% acceptance rate for SARP, which matches the rates described in the literature, despite the wide variation observed [7, 8, 14, 17]. Nygren et al. reported the importance of maximizing adherence to ERAS™ protocols, but this adherence is often hampered by their complexity [7, 14]. Some pilot studies describe more than 90% acceptance [8], while others observe very low rates of adherence to accelerated recovery programs [17]. The present study, as is the case with a few others described in the literature [18, 19], aims to simplify the protocol, adapting it to the actual circumstances of our institution. In this way, a simplified protocol was adopted (Table 1) and, even though it counted only seven items, the success rate remained at 60%. This result demonstrates that four out of ten patients did not benefit from SARP, making it clear that it is still possible to improve the cost–benefit ratio by increasing the acceptance rate of these protocols. The identification of these patients, as well as the reasons for non-tolerance, certainly makes it possible to achieve even better results in the future.

Development of postoperative complications, need for ostomy, a prolonged operative time, and surgeries with rectal resection, were associated with non-tolerance to the program in the univariate analysis. In the multivariate analysis, only development of complications, the need for ostomy, and a prolonged operative time remained as independent factors for a poor adherence to SARP.

Of the patients who had complications, 81% did not tolerate SARP (*p* < 0.0001). The possible reasons associated with non-tolerance to the program are immobilization over an extended period, greater difficulty in accepting a diet due to a longer ileum recovery, and uncertainty of being discharged after the complication.

Patients who required ostomy had a significantly higher SARP failure rate. Only 14.3% of patients with ostomy tolerated SARP (*p* < 0.0001), which matches the results from other studies that analyzed this finding [20, 21]. According to Delaney et al [22], the need for ostomy is an independent risk factor for prolonging hospital stay after colorectal surgery. Some studies have shown that setting up an educational program prior to discharge, with guidelines on how to care for the stoma, can shorten the hospital stay. These educational programs are even more effective if performed in the preoperative period, particularly with the involvement of a specialized physician or a stomatherapist [23–25]. Therefore, educational programs are an excellent investment of resources, in order to optimize the acceptance of accelerated postoperative recovery protocols.

Prolonged operative time was another factor that influenced the acceptance of SARP. The mean surgical time in the group that tolerated SARP was significantly lower than the mean in the group that did not. Adoption of measures that reduce surgery length, such as the strict standardization of the surgical technique and the participation of a senior surgeon in surgeries during the learning period, can not only contribute to reducing surgery time, but may also positively influence the implementation of SARPs.

In univariate analysis, another factor that significantly influenced the acceptance of the program was the type of surgery. Patients who underwent rectal surgeries had worse tolerance to the program than those who underwent colectomies. In patients who underwent colectomies, the acceptance rate of SARP was 78.6%, while in patients who underwent a rectal resection, the acceptance rate decreased to 14.3%. The ERAS Compliance Group [26] also reported poor results in patients who underwent rectal surgery, with a lower rate of adherence to the protocol, longer hospital stays, and higher readmission rates.

However, as demonstrated by multivariate analysis, the type of surgery itself was not statistically related to tolerance or non-tolerance to the implementation of the program. Nevertheless, patients who underwent rectal resections met the conditions associated with the worst acceptance of the SARP. In other words, they had longer surgeries, more postoperative complications and required ostomies more frequently, which were all variables that were significant in multivariate analysis. Prospective randomized studies analyzing the impact of these and other variables in the acceptance of the accelerated recovery protocols will help to identify the conditions that lead to failure in up to 40% of patients [5–8]. As the multidisciplinary team addresses these specific issues, the cost–benefit analysis of such programs may improve.

Other variables that were analyzed, such as age, BMI, sex, and type of disease (whether benign or malignant), did not influence the acceptance of SARP. These data demonstrate that SARPs can be adopted even in elderly patients and in those with high BMI, as demonstrated previously by the study of the ERAS Compliance Group [26], which found no differences between these variables in the acceptance of the ERAS protocol.

The ERAS™ philosophy has gained wider acceptance in the scientific community [27]. Recently, the ERAS Society [16, 28], as well as the American Society of Colon and Rectal Surgeons and Society of American Gastrointestinal and Endoscopic Surgeons, published the guidelines for clinical practice for enhanced recovery after surgery in colorectal surgery [29]. In a meta-analysis of sixteen randomized, controlled studies, Greco et al. [30] demonstrated that hospital stays were shortened by 2.28 days and that the complication rate decreased by 40% with the adoption of accelerated postoperative recovery programs. Moreover, Lee et al. [4], among other authors, [31, 32], found that ERAS™ allowed patients to get back to work sooner and to be less dependent on caregivers, without impairing quality of life.

In Brazil, in a study with over 5,000 patients who underwent large abdominal surgeries, Bicudo-Salomão et al. [33] demonstrated that operation costs, length of hospital stay, and complication rates were reduced in patients submitted to the ACERTO™ accelerated recovery protocol, compared to patients submitted to conventional postoperative care.

However, for the programs to succeed, it is essential that measures be well-tolerated by the patients [34, 35]. In that regard, this study was able to identify variables that negatively influence patients’ acceptance of SARPs.

### Strengths

It is randomized controlled trial. A simplified protocol of accelerated postoperative recovery was used, which can be easily implanted.

### Limitations

The main limitations of the present study were a reduced number of patients for the comparative analysis of some variables, such as rehospitalization and reoperation rates, use of a single criterion (length of hospital stay) to define the success of the adopted program, and a lack of individual analysis of the acceptance or non-acceptance of each variable of the program.

## Conclusions

The adoption of an accelerated recovery program, albeit simplified and focused on postoperative measures, was safe and brought benefits to patients tolerant to the program*.* In this study, the success rate in the implementation of the simplified accelerated recovery program was 60%.

The development of complications, prolonged operative time, and the need for ostomy were variables that negatively impacted the success rate in the implementation of SARP. Randomized prospective studies are necessary to define the role of these and other possible variables in the non-tolerance by patients to accelerated recovery programs.

Therefore, the adoption of specific and targeted measures to reduce the negative impact of these variables on the acceptance of SARPs can lead to better acceptance by specific groups of patients, improving results and making them more cost-effective.

## Data Availability

The datasets generated and/or analysed during the current study are available in http://www.ensaiosclinicos.gov.br/rg/RBR-2b4fyrand from the corresponding author on reasonable request.

## Abbreviations

ACERTO™: Accelerated recovery protocol
ASA: American Society of Anesthesiologists
BMI: Body mass index
CC: Conventional postoperative care
DC: Dindo–Clavien
ERAS™: Enhanced recovery after surgery
ICU: Intensive care unit
POD: Postoperative day
Q1; Q3: Interquartile range
SARP: Simplified accelerated recovery protocol
SD: Standard deviation
TME: Total mesorectal excision
